# Assessment of morphology, radiopacity gradient and mechanical properties of articular cartilage with contrast-enhanced high-resolution peripheral quantitative computed tomography

**DOI:** 10.3389/fbioe.2026.1756342

**Published:** 2026-06-26

**Authors:** Simone Fantoni, Matteo Berni, Roberta Fognani, Giulia Fraterrigo, Paolo Cardarelli, Fabio Baruffaldi, Massimiliano Baleani

**Affiliations:** 1 Laboratorio di Tecnologia Medica, IRCCS Istituto Ortopedico Rizzoli, Bologna, Italy; 2 Laboratorio di BioIngegneria Computazionale, IRCCS Istituto Ortopedico Rizzoli, Bologna, Italy; 3 Istituto Nazionale di Fisica Nucleare (INFN), Division of Ferrara, Ferrara, Italy

**Keywords:** articular cartilage, contrast agent, indentation, mechanical properties, X-ray imaging

## Abstract

**Introduction:**

Degenerative alterations in proteoglycans content and collagen structure compromise the mechanical integrity of articular cartilage and, therefore, its viscoelastic properties. Early detection of these subtle alterations is a prerequisite for enabling intervention with promising treatments under development. Although magnetic resonance imaging provides valuable compositional information, its limited spatial resolution restricts its effectiveness in evaluating thin, soft tissues such as articular cartilage. Contrast-enhanced high-resolution peripheral quantitative computed tomography (HR-pQCT) offers an optimal compromise between spatial resolution and radiation exposure, enabling high-resolution evaluation of the cartilage layer and potentially overcoming this limitation. This study investigated the feasibility of using HR-pQCT enhanced with the cationic contrast agent CA4+ to determine the proteoglycan gradient and thickness of articular cartilage from bovine stifle joints.

**Methods:**

The accuracy of thickness measurements was assessed by comparison with micro-computed tomography (μCT) measurements. It also investigated whether the local CT number (mean HU value of the articular cartilage, either uncorrected or corrected to the HU value of CA4+ bath) correlates with tissue mechanical competence. The latter was evaluated in terms of instantaneous, stress-relaxation and equilibrium responses by indenting the cartilage layer with a spherical indenter up to 15%, a value representative of the level of tissue deformation occurring *in vivo*.

**Results and discussion:**

A depth-wise gradient of CT number was distinguishable within the articular cartilage in contrast-enhanced HR-pQCT, allowing in post-processing the subdivision of cartilage into its three main layers. Additionally, contrast-enhanced imaging enabled the accurate quantification of articular cartilage thickness. Significant relationships of varying strength, assessed using Spearman’s rank correlation coefficient (ρ), were found between thickness and instantaneous elastic response (ρ = −0.85), stress-relaxation behaviour (ρ = −0.61), and equilibrium elastic response (ρ = −0.38) of articular cartilage. The same relationship with CT number showed comparable strength. These results, achieved through investigation of healthy bovine articular cartilage, support extending the proposed approach to human articular cartilage. The proposed approach, based on high-resolution X-ray scanner imaging enhanced with a cationic contrast agent, may have the potential to discriminate early-degenerated tissue by capturing subtle local alterations in thickness or CT number gradients. This could reveal changes in the relative thicknesses of the superficial, middle, and deep layers, across the articular surface, while also providing an indication of local alterations to the mechanical competence of the articular cartilage.

## 1 Introduction

The biphasic nature of articular cartilage (AC), consisting of a solid and a fluid phase, confers it unique mechanical properties, including compressive and tensile stiffness (Sophia Fox et al., 2009). The solid phase results from the non-uniform spatial arrangement of collagen bundles intertwined with proteoglycans (PGs). The collagen bundles show a distinctive layered organization, running tangentially to the articular surface in the superficial zone and becoming perpendicular in the deep zone (Muir, 1983). Within this bundle network, PGs are distributed according to a characteristic concentration gradient that increases towards the deeper layers (Muir, 1983). The core proteins of PGs are attached to one or more glycosaminoglycan (GAG) chains, responsible for the negative fixed charge density (FCD) in the solid phase (Roughley and Lee, 1994). FCDs create electrostatic repulsive forces that increase the compressive stiffness of the solid phase and interact with the fluid phase. The fluid phase is mainly composed of water and positively-charged ions which can flow through the solid phase (Setton et al., 1993).

The response of AC to any local stimulus depends on the integrity of the tissue. Any degeneration of AC leads to progressive alterations in its mechanical competence, ultimately resulting in joint dysfunction and pain (Setton et al., 1999). Since AC has a low cell density and lacks blood vessels, its self-repair ability is very limited. Therefore, it is crucial to be able to detect subtle changes in AC to: (i) enable the early identification of tissue degeneration, which could enhance the effectiveness of regenerative and pharmacological treatments (Roseti et al., 2019; Tangkanjanavelukul et al., 2025; Tschon et al., 2026), and (ii) accurately evaluate treatment outcomes (Ren et al., 2023).

Neglecting disease-associated biomarkers (Pickarski et al., 2011; Ji et al., 2019; Sandhu et al., 2023; Sundaram et al., 2025), hallmark indicators of AC degeneration include PGs loss (Buckwalter et al., 2005; Ojanen et al., 2018), superficial fibrillation (Pritzker et al., 2006), and thickness variation (Salzlechner et al., 2026). These compositional or morphological alterations impact the tissue mechanical response under loading, resulting in altered frictional properties and load-bearing capacity (Mäkelä et al., 2015; Fischenich et al., 2017; Salehuddin et al., 2025). These early signs can be detected by techniques involving an arthroscopic approach, such as ultrasound arthroscopy (Liukkonen et al., 2013; Penttilä et al., 2015) or near-infrared spectroscopy (Prakash et al., 2019; Linus et al., 2023). Despite these approaches can discriminate between healthy and pathological state in a clinical setting (Lee et al., 2008; Maeguchi et al., 2018; Zhang et al., 2024), the sensitivity of ultrasound-based analysis decreases because errors in cartilage thickness estimation can exceed 1 mm (Naredo et al., 2009; Maeguchi et al., 2018). Magnetic resonance imaging (MRI) can provide morphological information about articular cartilage as well as quantitative parameter correlated with tissue quality, but cartilage thickness tends to be overestimated with clinical scanners (up to 3T) due to the spatial resolution achievable, inducing partial volume effects (Koo et al., 2009; Schmitz et al., 2017; Bairagi et al., 2023). Despite this limitation, quantitative MRI techniques have the potential to detect tissue changes associated with early-stage osteoarthritis. Neglecting sophisticated approaches such as sodium nuclear magnetic spectroscopy (Zbýň et al., 2014; Zaric et al., 2021), chemical exchange saturation transfer imaging (Kogan et al., 2013; Wei et al., 2017; Dou et al., 2019) or diffusion tensor imaging of AC (Ferizi et al., 2017; Duarte et al., 2019), variable-strength correlations have been reported between GAG content and T_1_ρ relaxation time (Li et al., 2011; van Tiel et al., 2016; Jamaria et al., 2025) or delayed gadolinium-enhanced MRI (dGEMRIC) T_1_ (van Tiel et al., 2016), as well as between T_2_ relaxation time and water content (Chou et al., 2009) and collagen integrity (Ho et al., 2016). In contrast, relationships between MRI parameters and cartilage mechanical properties appear less straightforward (Svärd et al., 2018; Namiranian et al., 2020), with moderate-to-strong correlations reported only after data clustering (Hatcher et al., 2017; Hananouchi et al., 2023) or transformation, e.g., logarithmic transformation (Bae et al., 2024).

A potential alternative is contrast-enhanced X-ray imaging, which can provide superior spatial resolution compared to MRI. This approach enhances the X-ray visibility of targeted soft tissue components by employing radiopaque contrast agents (CAs). The CAs differ in their high-atomic number element incorporated, osmolality, pH, and molecular charge. Regardless of the CA type, the net molecular charge is the crucial feature determining the distribution of the CA within the tissue. It has been established that cationic CAs provide superior imaging results due to the electrostatic attraction exerted by FCD of GAGs (Stewart et al., 2017).

A promising CA is an experimental iodine-based cationic CA (CA4+) developed to enable quantitative assessment of PGs content within AC, which contributes to the mechanical properties of the tissue (Canal Guterl et al., 2010). (Lakin et al., 2013) and Stewart et al. (Stewart et al., 2017) found a strong positive correlation between contrast-enhanced tissue attenuation and compressive modulus. However, in both studies the imaging was performed using micro-computed tomography (μCT), which cannot be used in clinical practice. When a similar investigation was performed using a clinical CT scanner, no correlations were found between the CA4+ concentration and instantaneous or equilibrium modulus (Orava et al., 2024). In that study, the differences between thickness measured using clinical CT were in some cases greater than 1 mm, suggesting that the spatial resolution of clinical CT may be insufficient to properly capture the contrast-enhanced AC layer. Indeed, (Nickmanesh et al., 2018), using a CT scanner with a higher resolution (XtremeCT), found a correlation between stiffness and attenuation of contrast-enhanced tissue. In that study, AC stiffness was defined as “*the ratio of peak normal load to normal displacement*”. Although this definition assumes a linear tissue response and, therefore, represents a simplified indicator of cartilage mechanical behaviour, the results suggest that increasing spatial resolution may enable the detection of relationship between CA4+ concentration and cartilage mechanical properties in clinical settings. High-resolution peripheral quantitative computed tomography (HR-pQCT) scanner represents an optimal trade-off between radiation dose and spatial resolution (Cerbone et al., 2025).

It can therefore be hypothesised that the spatial resolution achievable with HR-pQCT may be sufficient to capture tissue morphology and detect correlations between contrast-enhanced attenuation and cartilage mechanical properties. The aim of this study was to investigate whether AC imaging, obtained by a contrast-enhanced HR-pQCT clinical protocol, can capture relevant tissue features and whether contrast enhanced attenuation of AC is related to its viscoelastic properties, derived from indentation test.

## 2 Materials and methods

**FIGURE 1:**
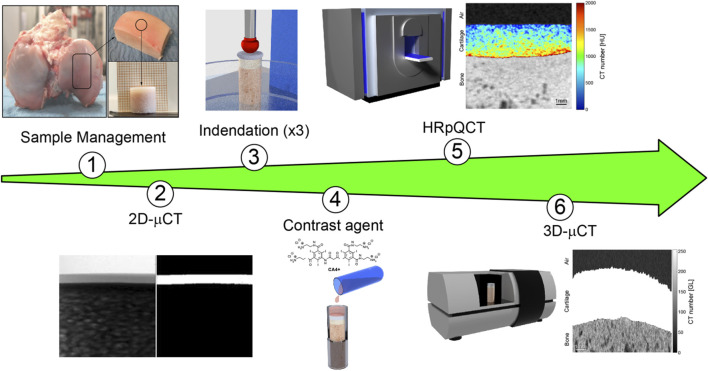
Workflow of the experimental pipeline.

The workflow of the study is reported in Figure 1.

### 2.1 Contrast agent

The CA employed by this study is CA4+, i.e., with formulation (5,50-[Malonylbis (azanediyl)]bis [N1,N3-bis(2-aminoethyl)-2,4,6-triiodoisophthalamide] chloride). One molecule of CA4+ includes six iodine atoms, and displays iodine fraction per molecule f = 0.5084, net positive charge q = +4, molecular weight mw = 1,354 g/mol. CA4+ salts synthesis was performed according to literature (Stewart et al., 2017). CA solution was prepared by dissolving CA4+ salts in phosphate-buffered saline (PBS) solution (1X, 7.4 pH, Life Technologies Europe B.V., Bleiswijk, The Netherlands) until reaching a concentration of 10 mgI/mL, effective to enhance significantly the X-ray attenuation of AC (Fantoni et al., 2022). The pH of the CA solution was balanced by adding NaOH 4M, i.e., up to a value of 7.4. The osmolality of PBS 1X and CA solution was measured with an osmometer (TypM 10/25 μL, accuracy 1 mOsm/kg, Löser Messtechnik, Berlin, Germany), yielding 307 mOsm/kg and 419 mOsm/kg, respectively.

### 2.2 Osteochondral tissue samples

Fourteen fresh bovine stifle joints of skeletally mature bovines were obtained from a local slaughterhouse within 24 h post-slaughter for food production purposes. Two osteochondral (OC) cuboids (Figure 1) were retrieved from the tibio-femoral surfaces of each joint (one from the femur, and one from the tibia). Regions with different AC thicknesses were considered to capture the full spectrum of thicknesses (Li et al., 2005; Espino et al., 2014), therefore accounting for the natural variation in composition, structure (Esquisatto et al., 1997; Quinn et al., 2005) and mechanical properties of the tissue (Moore and Burris, 2015; Hamsayeh Abbasi Niasar and Li, 2023; Berni et al., 2024). OC cuboids were excised by using a circular saw equipped with a diamond-coated blade (TR60 and MDP200, respectively; Remet, Italy). The process was performed under continuous water irrigation, to avoid the dehydration and heating of the tissues. Four cuboids from the tibial articular surface were discarded due to macroscopic damages occurred during the process. Cuboids were then wrapped in gauzes soaked in PBS 1X and frozen at −20 °C (1st freezing cycle). On a different day, OC cuboids were thawed at 4 °C in PBS 1X for 1 hour. Cylindrical OC cores (10-mm in diameter and height) were excised from each cuboid by means of a computer numerically controlled milling machine (ProLight 2000; Light Machines Corporation, Manchester, NH, United States) equipped with a diamond-coated coring tool. Prior to coring, OC cuboids were spatially positioned and tilted to ensure that the retrieved cores had an AC surface as flat as possible and perpendicular to the core axis. A total of 48 OC cores – 28 and 20 from the articular surface of femur and tibia, respectively–were excised, and stored at −20 °C in a PBS 1X-soaked gauze until indentation test (2^nd^ freezing cycle).

### 2.3 Indentation test

Subgroups of six OC cores were thawed in PBS 1X at 4 °C overnight. The AC thickness of each OC core was measured prior to indentation. Each OC core was acquired by a previously developed X-ray µCT planar acquisition protocol, aiming to obtain a preliminary measure of the AC thickness, information mandatory to execute a deformation-based indentation test. Each OC core was placed first in a custom-made polymethyl methacrylate sample holder and, then, within a µCT system (SkyScan 1072, SkyScan, Aartselaar, Belgium). Four planar images–each acquired after a 90-degree rotation of the core–were obtained at a resolution of 11.5 µm (total scan time of about 1 min). X-ray imaging was performed in air without CA, as the contrast AC-air was sufficient to identify the layer of the tissue. AC thickness was calculated using a custom-made MATLAB code (MATLAB version R2022b, MathWorks, Natick, MA, United States). Briefly: (i) planar images were cropped to exclude the outer sample holder; (ii) in each planar image, AC layer was differentiated from mineralized tissue and air, thanks to the sharp difference in radiopacity; (iii) AC layer was binarized; (iv) pixels within binarized AC layer in the vertical direction were considered and counted limitedly to a 10 pixel-thick peripheral annulus of the AC layer; (v) from the calculated values over the four planar images, the median value of AC thickness was evaluated. Full details of the procedure have been published elsewhere (Berni et al., 2025).

The six OC cores were then placed in a custom-made polyacetal sample holder with six cavities, each having an inner diameter of 10 mm and a depth of 20 mm. Prior to the insertion, polymethyl methacrylate was poured into the bottom of each cavity to secure the OC core, and a silicone-based grease was applied around the entire circumference of each OC core to prevent leakage of aqueous solution from the top AC surface to the lateral edges of the specimen. Then, PBS 1X (0.5 mL) was poured on the top surface of the OC cores to maintain the AC wet during testing.

The sample holder was mounted on a testing machine (Mach-1 V500css, Biomomentum Inc., Laval, QC, Canada). Each OC core underwent preconditioning, i.e., a preliminary indentation, followed by three indentations, each performed after a 40-min resting period. The extent of the resting period was confirmed by preliminary measurements (data not reported), as well as the relative literature (Jin and Lewis, 2004), also considering the insights of *in vivo* studies (Harkey et al., 2018; Cutcliffe et al., 2020; Park et al., 2024).

The indentation test involved applying a maximum nominal deformation equal to 15% of AC thickness by a 6 mm spherical indenter, at a deformation rate of 15%/s, perpendicular to the articular surface and at the centre of each OC core. The initial contact between the spherical indenter and the AC surface was defined using a load-based criterion, i.e., when the load reached 70 mN, corresponding to 20 times the resolution of the load cell used in the experiment. The nominal deformation was held for 300 s (Mohammadi et al., 2022) to assess the time-dependent behaviour of the stress. This testing protocol was used for both the preliminary indentation and the three indentations employed to determine the mechanical properties of AC. As for the three indentations, the protocol was employed to investigate the following parameters: the instantaneous elastic modulus (E_0_), which represents the ability of AC to withstand deformation when suddenly loaded, before interstitial fluid flow can occur; the time constant (τ) and the stretching parameter (β) of the load–time relaxation curve, which quantify how fast the transient viscous response of the tissue is exhausted; and equilibrium modulus (E_eq_), which represents the ability of AC to withstand deformation once the applied load is balanced and no further fluid flow occurs (Petitjean et al., 2023; Berni et al., 2024; Karpiński et al., 2025). Full details regarding the indentation protocol and the computation of these parameters have been published elsewhere (Berni et al., 2022). In addition to the experimental parameter derived from the trend over time of the applied load and tissue deformation, two further derived parameters were considered. Rather than treating the two parameters (τ and β) of the stretched exponential function independently, the exponent of the stretched exponential function – (1/τ)^β^–was computed, as it better represents the stress-relaxation behaviour (June and Fyhrie, 2008). The ratio between E_0_ and E_eq_ was also computed, as this better represents the contribution of the two phases to the AC elastic response over the solid matrix alone (Juras et al., 2009; Kumar et al., 2018).

### 2.4 HR-pQCT imaging

After completing the series of indentation tests, the PBS solution previously added to each cavity was replaced with 0.5 mL of CA solution. Following a 22-h rest at a room temperature (22 °C ± 2 °C) to allow the diffusion phenomena to reach equilibrium (Fantoni et al., 2022), the subgroup of six OC cores was acquired using an HR-pQCT system (XtremeCT II, SCANCO Medical AG, Brüttisellen, Switzerland). After removing the CA solution from the top surface of each OC core, the sample holder was placed within HR-pQCT by aligning the axes of the OC cores with the scanning axis. The OC cores were scanned using the following parameters: X-ray tube voltage = 68 kV, current = 1.47 mA, filtration = 1 mm Al + 0.2 mm Cu, pixel size = 60.7 μm, scan time = 6 min. The removed CA4+ solution was collected in isolated cuvettes and acquired in the same imaging session. Eventually, one cuvette of pristine CA4+ solution prepared for the experiment was acquired. At the end of the scanning procedure, OC cores were stored at −20 °C in separate sealed containers until the next assessment.

HR-pQCT images were reconstructed using a built-in Feldkamp-Davis-Kress reconstruction protocol. The reconstructed volumes were processed using a custom MATLAB script to estimate AC thickness and the depth-dependent distribution of CA4+ throughout the tissue, expressed as an attenuation—CT number (HU)—profile. Briefly, (i) from the reconstructed volume, single OC cores were virtually extracted by selecting cylindrical VOIs enclosing the entire OC sample; (ii) for each VOI, the interface between cartilage and the underlying bone tissue was considered for the thresholding procedure. The evaluation of the histogram of this interface region allowed to determine a thresholding value of CT number using the Otsu algorithm. CT numbers lying above this thresholding value were associated to bone tissue and therefore were set to zero; (iii) the interface between cartilage and overlying air was considered to implement a thresholding procedure based on CT numbers, again using Otsu algorithm applied to the resulting histogram. Tissue thickness was determined from the binarized AC volume by probing the volume with parallel columns of voxels oriented along the vertical direction (i.e., perpendicular to the reference surface). The height of each column was calculated. The mean value of these heights was used as a measurement of AC thickness.

Finally, the attenuation profile was determined for each vertical voxel column as a function of the distance from the AC–bone interface, after realigning the columns of voxels to a planar surface via translation. The mean intensity profile of each sample was calculated by averaging the CT numbers at each voxel height level. To determine the relative thicknesses of the superficial, middle, and deep layers, changes in the CT number gradient of the radiopacity profile–i.e., transition in slope–were identified and associated with two CT number values. These values served as thresholds for identifying the interfaces between the three layers. For each layer, the mean thickness was calculated using the procedure adopted for the entire cartilage layer. The mean CT number of the voxels within the segmented cartilage volume was also calculated. Similarly, the mean CT number of the voxels within the segmented volume of pristine and exhausted CA4+ – i.e., the CA solution before and after 22 h of contact with AC, respectively–was also calculated. The latter were then subtracted from the previously calculated mean cartilage CT number (ΔCT number) to account for the possible effect of progressive CA4+ dilution on its effectiveness in enhancing tissue radiopacity.

### 2.5 µCT imaging

On the day of the acquisition, OC cores were thawed at room temperature (22 °C ± 2 °C). Before the µCT acquisition, each OC core was exposed to the same CA solution previously used (see paragraph 2.3 and 2.4). This was done to avoid altering the radiopacity of the tissue, which could otherwise be affected by the use of PBS 1X or a fresh CA solution, while avoiding AC dehydration during the µCT scan. The OC cores were scanned using the following parameters: X-ray tube voltage = 50 kVp, current = 200 mA, filtration = 1 mm Al, pixel size = 11.5 μm, scan time = 60 min. Binarization of cartilage volume and measurement of AC thickness followed the procedure previously described (see Subsection 2.4).

### 2.6 Statistical analysis

Statistical analyses were performed using R software (R Core Team, 2025). The Kolmogorov–Smirnov test was used to analyse the distribution of AC thickness, mechanical parameters, and CT number. The difference between the AC thickness computed by HR-pQCT and µCT approaches (δ_t_) was evaluated using the Wilcoxon signed-rank test. Spearman’s rank correlation coefficient (ρ) was used to evaluate the strength of the relationships between AC thickness or CT number and the mechanical parameters, as no assumptions were made regarding the form of the relationships. All these evaluations considered the median values of the mechanical parameters derived from the three indentations performed on each OC core.

## 3 Results

**FIGURE 2:**
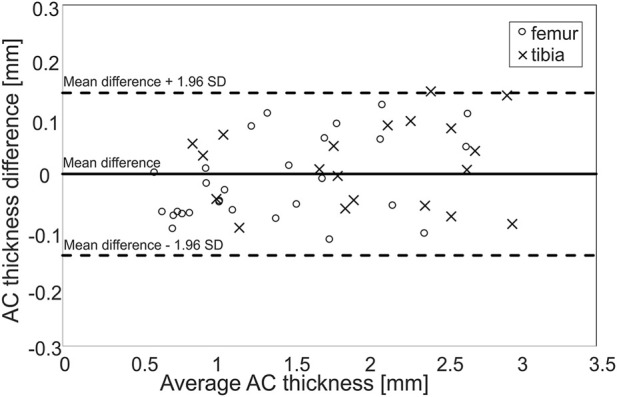
Bland-Altman plot of paired measurements of AC thickness, performed using μCT and HR-pQCT, following the contrast-enhancement of the tissue. All experimental points–except for one sample–lie between the agreement limits (dashed lines), defined as the mean difference (solid line) ± 2σ. Experimental points were distinguished whether the sample was extracted from a femoral condyle (○) or a tibial condyle (×).

The AC thickness of OC cores computed by HR-pQCT and µCT approaches was in the same range of 0.6–3.0 mm. No significant difference was found between the two groups (Wilcoxon signed-rank test, *p* = 0.86). The median value of δ_t_ found to be less than 0.01 mm (with 25th and 75th percentile being −0.06 mm and 0.07 mm, respectively). The agreement between the two measurement techniques is shown in the Bland-Altman plot (Figure 2).

**FIGURE 3:**
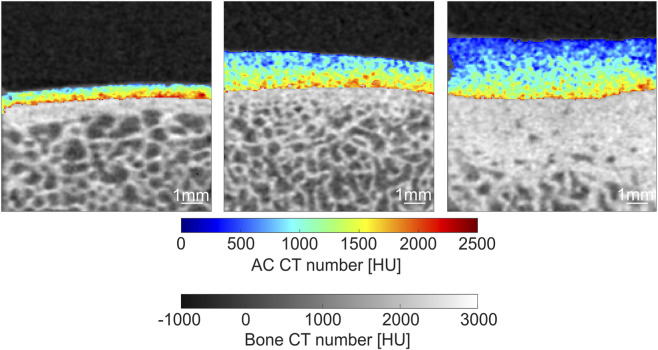
Sections of reconstructed volumes from HR-pQCT acquisitions. Representative samples of thin (left), average (centre) and thick (right) AC are shown, displaying the radiopacity of contrast-enhanced tissue.

**FIGURE 4:**
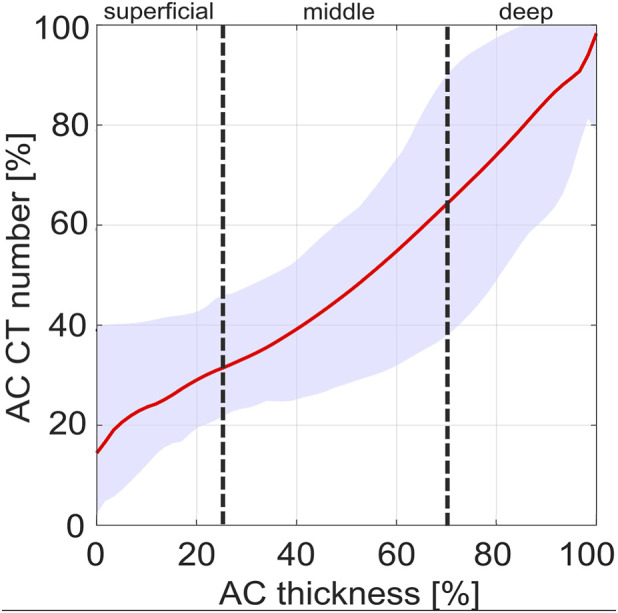
Average profile (red line) of radiopacity, evaluated across 48 OC cores previously normalised to their maximum value of CT number. The depth of each sample was also normalised to its AC total thickness. The grey area represents the range between the minimum and maximum values of the normalised profiles among all samples included in the analysis. The average profile points out an increasing radiopacity from the articular surface (depth = 0%) towards the deep layer (depth = 100%) of the AC. The vertical dashed lines represent the interfaces between the three constitutive layers of the AC.

**FIGURE 5:**
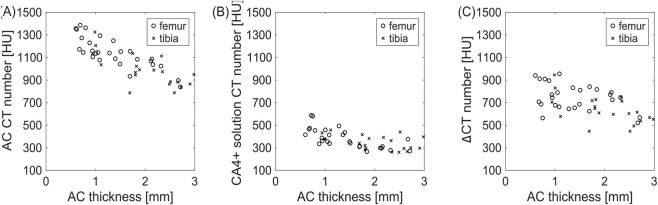
Scatterplots reporting the relationship between the AC thickness and the mean value of the CT number from different measurements, measured for the 48 samples. **(A)** Scatterplot of CT number of AC, evaluated as the mean value in the segmented volume of each sample. **(B)** Scatterplot of CT number of exhausted CA4+, evaluated as the mean value in the segmented volume of each cuvette. **(C)** Scatterplot of ΔCT number, evaluated as the difference between the CT number of AC and CT number of exhausted CA4+. Experimental points were distinguished whether the sample was extracted from a femoral condyle (○) or a tibial condyle (×).

Contrast-enhanced HR-pQCT imaging of AC revealed a consistent gradient in the distribution of CT numbers within the tissue (Figure 3). When normalised to their maximum value found in the contrast-enhanced tissue, the CT numbers exhibited a monotonic increase in attenuation from the articular surface to the deep zone of AC (Figure 4). An inverse relationship (ρ = −0.84, *p* < 0.001) was found between the mean CT number of the AC volume and the AC thickness. A similar relationship (ρ = −0.63, *p* < 0.001) was also found between the mean CT number of the exhausted CA4+ volume and AC thickness, which affects the relationship between the ΔCT number and AC thickness (ρ = −0.56, *p* < 0.001) (Figure 5).

**FIGURE 6:**
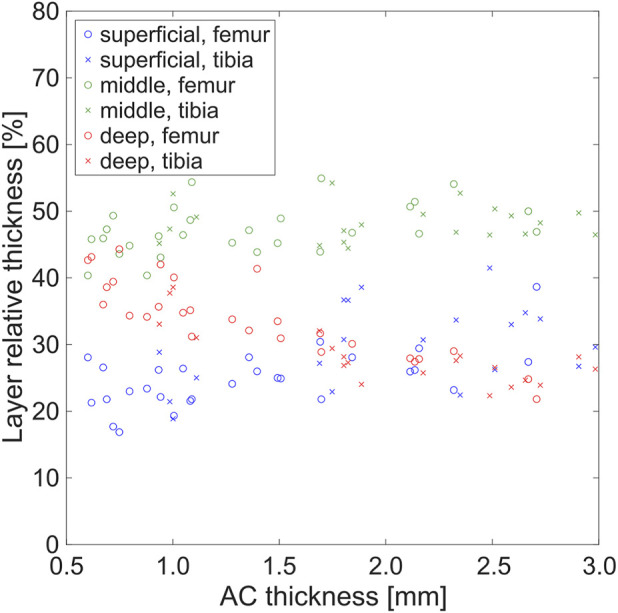
Scatterplot reporting the relationship between the total AC thickness and the mean value of layer relative thickness (evaluated as the ratio between the layer thickness and the total AC thickness), evaluated for the 48 samples. Different colours were assigned to each layer–namely, blue, green and red to superficial, middle and deep layer, respectively. Experimental points were distinguished whether the sample was extracted from a femoral condyle (○) or a tibial condyle (×).

The analysis of the attenuation profile allowed to determine two changes in attenuation profile, with variation in slope corresponding to 35 HU/voxel and 45 HU/voxel, for the superficial-to-middle layer and the middle-to-deep layer interfaces. These changes allowed to compute the mean relative thicknesses of the three AC layers: 25% (17%–42%), 45% (40%–55%), and 30% (22%–45%) for the superficial, middle and deep layers, respectively (Figure 6 **)**. For each layer, the CT number values – mean (min-Max) – were determined: 645 HU (402–992 HU), 991 HU (649–1281 HU) and 1439 HU (1,138–1753 HU) for the superficial, middle and deep layers, respectively. The CT number of pristine CA4+ solution yielded 620 ± 10 HU.

A total of 144 indentation tests were performed, with the preconditioning indentation excluded from the count. All 144 tests were considered valid, as (i) no discontinuities or unexpected trends were observed in the force–displacement curve during each indentation, and (ii) the AC response was repeatable across the three repetitions. Repeatability was assessed by calculating the coefficient of variation for each mechanical parameter (E_0_, τ, β, and E_eq_) based on three indentation tests per OC core. The average coefficients of variations were 2.9%, 2.0%, 0.6% and 2.7% for E_0_, τ, β and E_eq_, respectively. Out of 144 only one had a coefficient of variation greater than 10%. This case (13.2%) refers to an E_eq_ value. The median and range of the mechanical parameter values were as follows: E_0_: 2.9 (0.6–11.2) MPa; (1/τ)^β^: 0.530 (0.344–0.950) s^-1^; E_eq_: 0.2 (0.1–0.6) MPa; E_0_/E_eq_: 9.9 (3.9–64.5).

**FIGURE 7:**
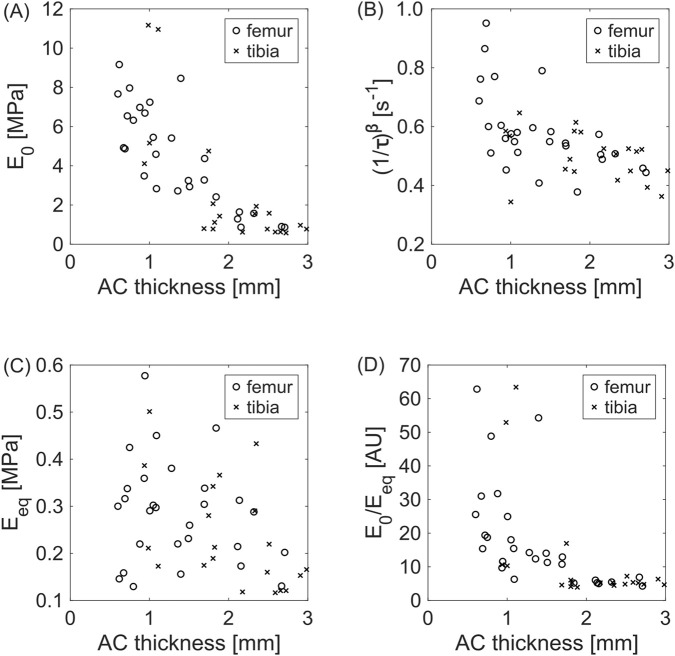
Scatterplots reporting the relationship between **(A)** the AC thickness and the median value of instantaneous modulus E_0_, **(B)** the median value of exponent (1/τ)^β^, **(C)** the equilibrium modulus E_eq_ and **(D)** the median value of E_0_/E_eq_, evaluated for the 48 samples. Experimental points were distinguished whether the sample was extracted from a femoral condyle (○) or a tibial condyle (×).

**FIGURE 8:**
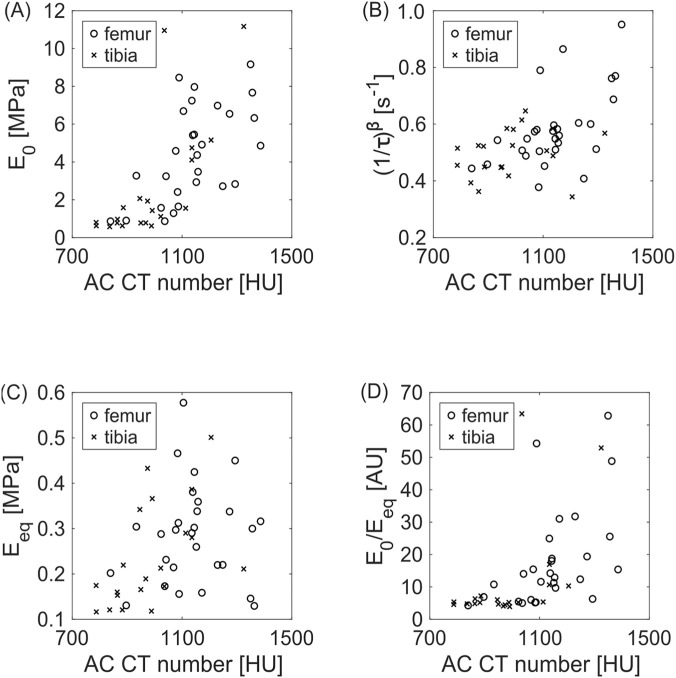
Scatterplots reporting the relationship between AC CT number and **(A)** the median value of instantaneous modulus E_0_, **(B)** the median value of exponent (1/τ)^β^, **(C)** equilibrium modulus E_eq_, and **(D)** the median value of E_0_/E_eq_, evaluated for the 48 samples. Experimental points were distinguished whether the sample was extracted from a femoral condyle (○) or a tibial condyle (×).

**FIGURE 9:**
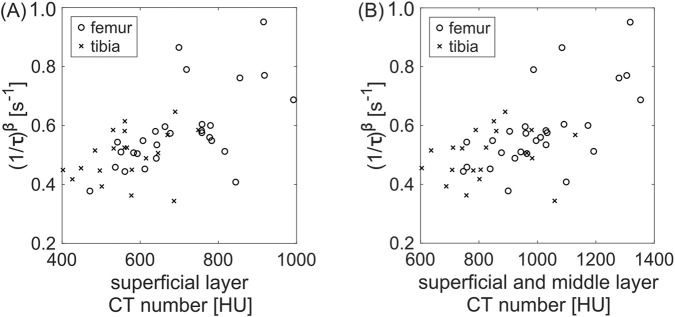
Scatterplots reporting the relationship between CT number evaluated for the superficial layer **(A)** and the superficial and middle layers combined **(B)**, and median value of exponent (1/τ)^β^, the parameter β should be reported as exponent of the part (1/τ), evaluated for the 48 samples. Experimental points were distinguished whether the sample was extracted from a femoral condyle (○) or a tibial condyle (×).

All four parameters showed a significant relationship with both the AC thickness and CT number, computed by HR-pQCT approach for superficial layer, superficial-middle layer and full AC thickness. The strength of these relationships depends on the considered mechanical property. ρ values are resumed in Table 1. The scatterplots of the strongest relationships of Table 1, as well as all those referring to the parameter (1/τ)^β^, are reported in Figures 7–9.

**TABLE 1:** ρ values calculated for the relationships between AC thickness–superficial layer, superficial-middle layer, and full–or CT number value, both computed by HR-pQCT approach, and viscoelastic properties–the instantaneous elastic modulus E_0_, the exponent of the stretched exponential function (1/τ)^β^, the equilibrium modulus E_eq_, and the ratio E_0_/E_eq_.

AC morphological feature or radiopacity	E_0_	(1/τ)^β^	E_eq_	E_0_/E_eq_
AC thickness	ρ = −0.85***	ρ = −0.61***	ρ = −0.38**	ρ = −0.79***
Superficial layer CT number	ρ = 0.66***	ρ = 0.57***	ρ = 0.14	ρ = 0.68***
Superficial-middle layer CT number	ρ = 0.73***	ρ = 0.53***	ρ = 0.32*	ρ = 0.69***
Full thickness CT number	ρ = 0.77***	ρ = 0.49***	ρ = 0.39**	ρ = 0.72***
Full thickness ΔCT number	ρ = 0.50***	ρ = 0.29 *	ρ = 0.38**	ρ = 0.41**

* meaning p < 0.05; ** meaning p < 0.01; *** meaning p < 0.001.

## 4 Discussion

The purpose of the present study was to investigate whether it is possible to capture relevant AC features by analysing contrast-enhanced imaging collected using HR-pQCT, as well as to explore possible relationships between mechanical properties and image-derived parameters.

Consistent agreement was observed between AC thickness measurements obtained using μCT and HR-pQCT approaches, which supports the reliability of HR-pQCT for evaluating AC thickness under contrast-enhanced conditions. The maximum discrepancy in the present dataset was approximately two and a half times the resolution of the HR-pQCT (∼150 μm), which is sufficient for detecting alterations in AC thickness in a clinical setting.

HR-pQCT images highlighted a depth-dependent distribution of CT numbers within AC. Bearing in mind the cationic nature of CA4+, the increase of CT numbers from the articular surface towards the deep layer of AC denotes an increment of PGs content through the thickness of the tissue (Honkanen et al., 2019; Fantoni et al., 2022). This evidence is consistent with existing literature, addressing the quantification of PGs using ex-vivo reference techniques – e.g., histology (Pastrama et al., 2019), histochemistry (Xia et al., 2008), digital densitometry (Mohammadi et al., 2022), Fourier transform infrared (Yin et al., 2011) and Raman spectroscopy (Shehata et al., 2023) – or imaging methods – e.g., contrast-enhanced X-ray (Lakin et al., 2013; Stewart et al., 2017; Honkanen et al., 2019; Bhattarai et al., 2021). The inverse relationship between AC thickness and the mean CT number value deserves to be highlighted. Although this relationship weakens when the mean ΔCT number is considered, it suggests that the PGs content decreases with increasing the AC thickness. This relationship has been poorly investigated in literature. To the authors’ best knowledge, (Kamisan et al., 2013) represents the only experimental study addressing this aspect, reporting a significant positive correlation between GAG content and AC thickness. However, it is important to note that this result was obtained by evaluating AC samples retrieved from the femoral condyles of rats, rabbits, and goats. The apparent upward trend is largely influenced by three data points corresponding to samples from goat knee joints, which were combined with those from rat and rabbit cartilage. With reference to figure 4b of the cited study, when the data are clustered by species, the positive trend may no longer be evident. The inverse relationship between AC thickness and the mean CT number value suggests that, in a healthy AC, the three layers – the superficial, middle and deep layers – do not increase in thickness proportionally as the overall AC thickness increases. This hypothesis is further supported by the relative thickness values evaluated for the superficial, middle and deep layers of total AC thickness. The present findings suggest that the middle layer accounts for approximatively half of AC total thickness, whereas the superficial and deep layers show opposing trends as the AC thickness increases. These outcomes are consistent with those of a study that used stains with affinity for PGs to differentiate the constitutive layers of AC (Kiviranta et al., 1985). Nevertheless, it should be noted that these findings are not consistent with those reported in other studies that differentiated the AC layers using different criteria, i.e., cellular size, organization, and orientation (Pedersen et al., 2013), the preferential orientation axis of the sulphated glycosamino-glycan molecules (Arokoski et al., 1996), collagen network organisation (Kääb et al., 1998; Rieppo et al., 2003; Alhadlaq and Xia, 2004). This disagreement between the findings of different studies may be due both to the criterion used to determine the thickness of the AC layers, since the depth-dependent changes in the features being investigated may not be consistent, and to the local differences in the AC of the patella, femur or tibia.

Indentation tests were used to determine the viscoelastic properties of AC. The experimental values of the parameters measured in this study were consistent with those reported in the literature (Simha et al., 2007; Juras et al., 2009; Bonnevie et al., 2012; Moore and Burris, 2015; Joukar et al., 2023; Berni et al., 2022). The wide variation in the values of mechanical parameters was expected, given that the OC cores examined in the present study were harvested from different regions of the knee and the region-dependent mechanical properties of AC reported in previous studies (Seidenstuecker et al., 2019; Li et al., 2021; Hamsayeh Abbasi Niasar and Li, 2023). These variations reflect differences in tissue thickness, internal structure and composition across locations within the knee articular surface (Espino et al., 2014; Risch et al., 2021; Fischenich et al., 2022; Ma et al., 2024; Esquisatto et al., 1997; Li et al., 2005; Ariyachaikul et al., 2025; Quinn et al., 2005). All these factors contribute to the modulation of the AC response under applied loads. With reference to the parameters E_0_ and E_eq_ – representing the mechanical behaviour in absence of fluid flow –, previous studies indicate that the E_0_ value may be influenced by the overall thickness and internal structure of the AC, specifically the thickness of its constituent layers, as well as tissue composition, including the collagen matrix, GAG content, and fluid fraction (Arokoski et al., 1999; Korhonen et al., 2002; Julkunen et al., 2010; Joukar et al., 2023). All the mentioned factors also seem to contribute to the E_eq_ value, although to different extents (Armstrong and Mow, 1982; Treppo et al., 2000; Williamson et al., 2001; Bansal et al., 2010; Huttu et al., 2014; Joukar et al., 2023). Moving to the parameter (1/τ)^β^, this is related to the tissue stress-relaxation response. Based on the models proposed to predict this behaviour, AC thickness, tissue permeability, the elastic modulus at equilibrium, matrix viscoelasticity as well as strain induced in the tissue and extension of the compressed articular surface are all factors contributing to determine the stress-relaxation response (Mow et al., 1984; Williamson et al., 2001; Joukar et al., 2023). Therefore, the AC response to external loads is a complex phenomenon resulting from interactions among its specialized structural components, which should be considered when evaluating potential relationships between tissue features and mechanical parameters.

The relationships between AC thickness and mechanical response support the hypothesis that the thickness of healthy AC plays a crucial role in the manifestation of the time-independent response to indentation with a spherical indenter (Argatov et al., 2013). It is likely that all the previously mentioned factors contributed optimally to mechanical behaviour of healthy AC tissue, such as that tested in the present study. Therefore, under these conditions, cartilage thickness appears to be a primary factor influencing the instantaneous AC response, as measured by the parameter E_0_. This effect also persists when considering the parameter that captures the contribution of the two phases to the AC elastic response relative to the solid matrix alone (E_0_/E_eq_). However, when the equilibrium response (E_eq_) of AC is considered, the tissue composition appears to play a more important role than the AC morphology. Indeed, the relationship between E_eq_ and AC thickness is weaker than that between E_0_ and AC thickness and becomes comparable to that between CT number and E_eq_. This result is in agreement with a previous study, where a moderate correlation (ρ ∼ -0.4) between E_eq_ and AC thickness has been reported (Julkunen et al., 2009). It may be unexpected that the strength of the relationship between CT number and E_0_ is greater than that between CT number and E_eq_. This outcome can be explained by considering that i) CT number is strongly correlated with AC thickness, which in turn shows a strong association with E_0_. Consequently, a weaker relationship between CT number and AC thickness (Figure 7) results in a weaker relationship between CT number and E_0_, and (ii) although CT number is also related to PGs content, this relationship is comparatively weaker when the analysis is restricted to healthy tissue – i.e., excluding data from degenerated tissue (Bansal et al., 2010; Lakin et al., 2013). The present findings partially agree with data reported in previous reports. The instantaneous modulus E_0_ was found to correlate with the CT number of CA4+-enhanced cartilage in equine and murine species (Lakin et al., 2016; Nelson et al., 2019; 2021; 2024). The correlation between E_0_ and CT number presented here aligns with those of the cited works – ρ = 0.50–0.77 vs. r = 0.30–0.64. Two other previous works have addressed the correlation between the equilibrium modulus E_eq_ and CT number in AC retrieved from bovine stifle joint. The works of Stewart et al. and Lakin et al. provided linear correlations values of R^2^ = 0.54 (r = 0.74) and R^2^ = 0.90 (r = 0.95), respectively (Lakin et al., 2013; Stewart et al., 2017). Both the studies applied unconfined compression with stress relaxation. This testing configuration determines a flat deformation throughout the entire thickness instead of a localized deformation gradient, which likely correlates better with the CT number of the AC layer. Additionally, the latter study pooled data collected on healthy and experimentally-degraded AC which may have strengthened the correlation coefficient by broadening the range of the independent variable. These methodological differences may explain the weaker relationship found in the present study. However, we opted for the spherical and a clinical CT scanner because i) indentation testing allows mapping the mechanical properties across the articular surface (Seidenstuecker et al., 2019) and ii) clinical CT scanner enables imaging of the entire knee joint (Kroker et al., 2017), thereby enabling the application of the proposed approach to the entire articular joint surface. It is worth noting that the strength of the relationships decreased when moving from full-thickness CT number to superficial–middle layer CT number and, subsequently, to superficial layer CT number. This trend can be explained by the fact that the depth-dependent AC deformation profile is influenced by the indentation depth, indenter radius, contact radius, and AC thickness (Esteki et al., 2020). The instantaneous and equilibrium responses to indentation involve the entire AC, as the indentation test was performed using a 6 mm spherical indenter with a nominal deformation of 15% of the tissue thickness (Briant et al., 2015; Pastrama et al., 2022).

AC stress-relaxation is related to both fluid flow and the viscoelasticity behaviour of the solid phase. It has been demonstrated that AC stress-relaxation is influenced by both the charge concentration and valence of solution cations (June et al., 2009). This finding supports a role of FCD of the solid phase in the transient response of AC before equilibrium is reached. It has been suggested that FCD distribution also affects the tissue permeability (Maroudas and Bullough, 1968). Indeed, permeability is not homogeneous throughout the cartilage layer, being highest near the articular surface and progressively decreasing towards the deep zone (Chen et al., 2001). On the other hand, the collagen network also plays a role in the transient response (Federico and Herzog, 2008). During this phase, as deformation of the solid phase occurs, tissue permeability decreases (Mansour and Mow, 1976). Overall, the transient response is regulated by multiple factors that vary over time. This may explain the weak relationship (ρ = 0.29–0.49) found between the CT number and the exponent of the stretched exponential function (1/τ)^β^, which increased up to 0.57 when the CT number of the superficial layer is considered. This finding is partially supported by a previous report, in which only a moderate relationship (r = 0.59) was found between the CT number and the time constant τ (Nelson et al., 2019).

Several limitations should be acknowledged.

First, this study was performed on OC cores. OC core extraction exposed the AC to two freeze-thaw cycles. Previous studies have suggested that freezing-thaw cycles may affect the mechanical properties (Changoor et al., 2010; Qu et al., 2014; Peters et al., 2017), as well as the structure and composition (Qu et al., 2014) of AC. Although the assessment of biological tissues should ideally be performed in fresh condition, the design of the present study was implemented in order to standardize the management procedure for all the samples, thereby avoiding variability associated with differences in the timing of the experimental phases. Additionally, it could be argued that indentation testing performed on OC cores may provide biased results due to boundary effects. However, considering a sphere–plane Hertzian contact (Bushby and Jennett, 2000), and a maximum AC thickness of 3.0 mm (see the “Results” section), the maximum contact radius (a) is calculated to be 1.6 mm. Since the AC region affected by spherical indentation extends to less than 3a (Berni et al., 2022) – i.e. 4.8 mm, which is smaller than the radius of the OC cores used in this study –, boundary effects were unlikely to have significantly influenced the tissue response to indentation testing.

Second, the 22-h interval between the mechanical characterization of AC via indentation and the HR-pQCT scan may have led to some tissue degradation. However, this time frame was previously identified as the minimum duration required to achieve equilibrium in CA4+ diffusion within AC, and thus represents a prerequisite for reliable detection of CT number gradients.

Third, this study aimed to explore if the spatial resolution achievable with HR-pQCT may be sufficient to capture tissue morphology and detect correlations between contrast-enhanced attenuation and cartilage mechanical properties. For this reason, the analysis was limited to Spearman’s rank correlation test and did not include more complex approaches for evaluating the independent contribution of each predictor or accounting for potential confounding factors.

Fourth, the relationships between AC morphology, composition and mechanical properties found in this study are limited to healthy bovine tissues. These findings cannot be generalised to human AC, considering the difference in the main features of healthy tissue (Athanasiou et al., 1991; Nissi et al., 2007; Taylor et al., 2012; Pedersen et al., 2013; Rieppo et al., 2013; Temple et al., 2016) and, furthermore, the changes induced by pathologies, i.e., osteoarthritis, where reduced thickness and alteration in PGs content result in a progressive deterioration of the tissue mechanical competence (Kumar et al., 2018; Ebrahimi et al., 2022; Linus et al., 2024). Therefore, specific studies on human AC are warranted to properly elucidate these relationships.

Fifth, CT number normalisation was performed by subtracting the CT number of the exhausted CA4+ solution. While this approach partially realigns the CT number of the AC tissue, it cannot compensate for nonlinear diffusion effects, which may alter the described relationships. However, the aim of the present study was to investigate the exsistence of relationships between contrast-enhanced attenuation of AC and its viscoelastic properties, derived from indentation, to further support studies on human AC.

Taking these limitations into account, the present results suggest that contrast-enhanced HR-pQCT imaging can accurately determine the local thickness of AC. Additionally, grey-level gradients within the AC layer can be captured, enabling estimation of the relative thicknesses of the superficial, middle, and deep layers. Lastly, despite interspecies differences between bovine and human AC, it may be hypothesized that some relationships among AC thickness, local contrast-enhanced attenuation, and mechanical properties also exist in human AC. Therefore, this exploratory study, performed on the AC of bovine stifle joints, supports further investigation with the proposed approach to be performed on the entire human knee in a simulated clinical setting to verify the existence of the hypothesised relationships.

## 5 Conclusion

Contrast-enhanced HR-pQCT appears to be a promising approach for investigating key features of AC, allowing accurate measurement of its local thickness. Furthermore, the framework presented here enables the investigation of the relationships between AC thickness, local contrast-enhanced attenuation, and mechanical properties of the tissue, thereby providing further insight into the interplay between X-ray detectable features and the functional behaviour. The framework proposed in the study could be a valuable tool for evaluating the efficacy of therapeutic strategies and biomaterials designed to regenerate or repair AC, and for supporting the development of scaffolds better mimicking native tissue behaviour. In the perspective of implementing this approach into clinical practice, the safety of using CA4+ must be fully verified, and the potential of contrast-enhanced HR-pQCT should be further investigated in both healthy and pathological human AC.

## Data Availability

The datasets analysed for this study is accessible at Zenodo, https://doi.org/10.5281/zenodo.20038656.
